# Six-Year Retrospective Review of Hospital Data on Antimicrobial Resistance Profile of *Staphylococcus aureus* Isolated from Skin Infections from a Single Institution in Greece

**DOI:** 10.3390/antibiotics6040039

**Published:** 2017-12-20

**Authors:** Christina Stefanaki, Alexandra Ieronymaki, Theoni Matoula, Chrysseis Caroni, Evaggelia Polythodoraki, Stella-Eugenia Chryssou, George Kontochristopoulos, Christina Antoniou

**Affiliations:** 1 Dermatology Department, Andreas Sygros University Skin Hospital, 16121 Athens, Greece; matoula_th@yahoo.gr (T.M.); gkontochris@gmail.com (G.K.); Christinaantoniougr@yahoo.com (C.A.); 2 Microbiology Department, Andreas Sygros University Skin Hospital, 16121 Athens, Greece; alexandraieronymaki@gmail.com (A.I.); iponiki@live.com (E.P.); chryssoustella@gmail.com (S.-E.C.); 3 Department of Statistics and Mathematics, National Technical University of Athens, 15780 Athens, Greece; ccar@math.ntua.gr

**Keywords:** *Staphylococcus aureus*, MRSA, cefoxitin, mupirocin, fusidic acid, resistance

## Abstract

*Objective:* To determine the prevalence of resistant strains of *Staphylococcus aureus* (*S. aureus*) isolated from Skin and soft tissue infections (SSTI) to various antibiotics. *Material and Methods:* All culture-positive results for *S. aureus* from swabs taken from patients presenting at one Greek hospital with a skin infection between the years 2010–2015 were examined retrospectively. Bacterial cultures, identification of *S. aureus* and antimicrobial susceptibility testing were performed using the disk diffusion method according to Clinical and Laboratory Standards Institute (CLSI) guidelines and European Committee on Antimicrobial testing (EUCAST) breakpoints. EUCAST breakpoints were applied if no CLSI were available. *Results:* Of 2069 *S. aureus* isolates identified, 1845 (88%) were resistant to one or more antibiotics. The highest resistance was observed for benzylpenicillin (71.9%), followed by erythromycin (34.3%). Resistant strains to cefoxitin defined as MRSA (methicillin-resistant *S. aureus*) represented 21% of total isolates. Interestingly, resistance to fusidic acid was 22.9% and to mupirocin as high as 12.7%. Low rates were observed for minocycline, rifampicin and trimethoprim/sulfamethoxazole (SXT). Resistance to antibiotics remained relatively stable throughout the six-year period, with the exception of cefoxitin, fusidic acid and SXT. A high percentage of MRSA strains were resistant to erythromycin (60%), fusidic acid (46%), clindamycin (38%) and tetracycline (35.5%). *Conclusions:* Special attention is required in prescribing appropriate antibiotic therapeutic regimens, particularly for MRSA. These data on the susceptibility of *S. aureus* may be useful for guiding antibiotic treatment.

## 1. Introduction 

Antimicrobial resistance has become a global public health threat in recent years and is primarily driven by extensive and often unnecessary use of antibiotics [1,2,3]. *S. aureus* is among the most common pathogens of human beings and is the main pathogen implicated in skin and soft tissue infections, causing impetigo, folliculitis, furunculosis, cutaneous abscesses, cellulitis and infected eczematous dermatitis [4]. *S. aureus* resistance to antibiotics is growing and methicillin-resistant *S. aureus* (MRSA), once confined to individuals in the hospital setting, now appears routinely in the community [5].

Understanding the epidemiology of MRSA is crucial to establishing public health interventions and national hospital surveillance programs have long tracked MRSA. As antibiotic therapy, either topical or systemic, is the main treatment for skin infections, we conducted the present study to evaluate the antimicrobial susceptibility of *S. aureus* in our setting and to determine the prevalence of resistant strains.

## 2. Materials and Methods

“Andreas Sygros” University Hospital of Cutaneous and Venereal Diseases is a tertiary care referral center for skin diseases serving the population of Greater Athens, comprising four million people, as well as other parts of Greece. 

We retrospectively reviewed the files of the Microbiology Department of Andreas Sygros Hospital between 1 January 2010 to 31 December 2015 (6 years) and we collected all culture-positive results for *S. aureus* from swabs taken from patients presenting to the Hospital with a skin infection, either impetigo, furuncle, folliculitis or infected dermatosis. Clinical isolates were divided into those deriving from patients visiting the outpatient clinics and those from patients with leg ulcers who were visiting the Leg Ulcer Unit on a regular basis. Antimicrobial susceptibility of *S. aureus* isolates was recorded and resistance was defined as complete resistance to an antimicrobial agent. All available clinical data such as sex or site of specimen collection were recorded. For comparisons over time the patients were divided into four groups, depending on the year of presentation: group 1 = 2010 and first half of 2011; group 2 = rest of 2011 and 2012; group 3 = 2013; group 4 = 2014–2015. The study was approved by the Institutional Review Board. 

### 2.1. Microbiological Methods

Bacterial cultures were performed according to standard microbiological methods. Skin swabs for culture were taken from the lesions using sterile cotton tips. Samples were inoculated and cultured into blood agar plates. *S. aureus* isolates were identified on the basis of colony morphology, Gram stain, catalase and coagulase production and the Deoxyribonuclease (DNase) test.

Antimicrobial susceptibility testing was performed with the disk diffusion method according to Clinical and Laboratory Standards Institute (CLSI) guidelines for benzylpenicillin, cefoxitin, erythromycin, clindamycin, trimethoprim/sulfamethoxazole (SXT), rifampicin, gentamicin, minocycline and tetracycline [6], and according to the European Committee on Antimicrobial Susceptibility Testing (EUCAST) breakpoints for fusidic acid and mupirocin [7]. The minimum inhibitory concentrations (MICs) of vancomycin and teicoplanin were determined with a gradient method (Etest, bioMérieux, Marcy l’Etoile, France).

Methicillin-resistant *S. aureus* (MRSA) isolates were defined as those showing resistance to cefoxitin by the disk diffusion method [6].

### 2.2. Statistical Analysis

The prevalence (%) of resistance of *S. aureus* isolates to various antimicrobials was calculated. Incidence was compared between groups of patients or groups of isolates using Pearson’s chi-squared test of homogeneity in contingency tables, with Yates’ continuity correction in the case of 2 × 2 tables.

## 3. Results 

A total of 2069 *S. aureus* isolates were identified corresponding to the same number of different patients through the years 2010–2015. Group 1 comprised 612 isolates, group 2 477, group 3 393 and group 4 714. *S. aureus* was more often isolated from men (55.3–58.3%) throughout the whole study period. Overall, 1845 (88%) isolates were resistant to one or more antibiotics. Resistance to different antimicrobial agents is presented in Table 1. The highest resistance was observed for benzylpenicillin (71.9%) (meaning resistance to penicillinase-labile penicillins, including ampicillin, amoxicillin and ticarcillin) followed by erythromycin (34.3%). Resistant strains to cefoxitin defined as MRSA were 440 and represented 21% of total isolates. Interestingly, resistance to fusidic acid was found to be 22.9% and to mupirocin as high as 12.7%.

Resistance rates observed for minocycline, rifampicin and trimethoprim/sulfamethoxazole (SXT) were low (0.6%, 1.2% and 4.6%, respectively). Resistance to antibiotics remained relatively stable across the six-year period, with the exception of cefoxitin (*p* < 0.001), fusidic acid (*p* = 0.019) and trimethoprim/sulfamethoxazole (SXT) (*p* = 0.044) (Table 2). A drop in cefoxitin resistance and consequently in the prevalence of MRSA isolates was observed in 2013 (1.4%) for reasons that cannot be explained, whereas resistance was similar throughout the other years. All (100%) MRSA isolates were found resistant to at least one other antibiotic. A high percentage of MRSA strains were resistant to erythromycin (60.2%), fusidic acid (45.7%), clindamycin (38.9%) and tetracycline (35.5%) (Table 2). Interestingly, only 17.7% of MRSA isolates were resistant to mupirocin. 

No statistically significant difference was observed between males and females, as 87.4% of male patients and 88.9% of female patients had an isolate resistant to one or more antibiotics. *S. aureus* tended to be resistant to clindamycin (*p* = 0.021) and SXT (*p* = 0.003) if the sample was taken from the limbs and to mupirocin (*p* = 0.001) if the sample was taken from the face (Table 3). Only 86 isolates corresponded to patients attending the Leg Ulcer Unit. These demonstrated a similar resistance profile to outpatients, except that decreased resistance to tetracycline was identified compared to outpatient isolates (*p* < 0.001) (Table 4). 

## 4. Discussion

In our study we retrospectively reviewed antimicrobial resistance in a large number of *S. aureus* isolates and we found that a high percentage (88%) were resistant to one or more antibiotics. However, MRSA strains represented only 21% of the total isolates. MRSA incidence remained relatively stable throughout the six-year period with the exception of 2013, when a drop in cefoxitin resistance and consequently MRSA isolates was observed. Global MRSA rates vary from 26–30% in Africa, Europe and the Middle East to 50% in North America and 55% in Latin America, significantly decreasing overall between 2004 and 2014 [7]. In Greece, where antimicrobial drug resistance remains high, MRSA prevalence reaches 40%, which is among the highest in Europe [8,9,10,11,12]. However, in a multicenter European study investigating MRSA infections among patients in the emergency department, lower MRSA rates (27%) from Greece were reported, compatible with our results [13]. 

The multidrug-resistant phenotype is a particular characteristic of MRSA, related to the global presence and spread of multidrug-resistant clones [14,15]. Apart from the characteristic resistance to all b-lactams, MRSA also demonstrates resistance to many classes of antibiotics such as macrolides, clindamycin, fluoroquinolones, tetracycline, mupirocin, and trimethoprim/sulfamethoxazole (SXT) [4,16,17], and a high percentage of MRSA strains (31.1%) have been reported resistant to erythromycin [5]. Our study shows high resistance of *S. aureus* strains to erythromycin (34.4%), much higher for MRSA (60%), making macrolides an unsuitable first-line choice for treatment of skin infections. Similar (31.7%) [18] or higher (49%) [19] rates of resistance to macrolides have been reported from other studies performed in Greece. Clindamycin is a bacteriostatic agent that is favored in the setting of staphylococcal skin infection due to its excellent skin penetration [20]. Clindamycin and SXT require particular attention as they are recommended as first-line choices for outpatient treatment of SSTI [21,22]. Clindamycin resistance steadily increased over the last few decades reaching 10% in outpatient departments [23]. We found increased resistance for clindamycin (18%) but low for STX (4.6%), however 38% and 12.5% of MRSA isolates were resistant to clindamycin and STX, respectively. Our results are similar for clindamycin but much higher for STX compared with previous studies in Greek hospitals [12,18,19]. The high resistance to clindamycin limits its use for SSTI but STX may still be an option. 

According to our and previous results [12], rifampicin retains excellent activity against *S. aureus* infections, probably because of its limited use in the context of staphylococcal infections. Low rates of resistance were also found for minocycline in accordance with a large multi-center study [9] but resistance to tetracycline was found to be 17%, reaching 35.5% in MRSA.

No *S. aureus* isolates in our study were found resistant to vancomycin and few to teicoplanin. Similarly, no *S. aureus* isolates were found resistant to vancomycin in a large European multicenter study including Greece [7] and in a 12-year Greek study [14], making vancomycin particularly suitable for the treatment of severe and complicated SSTI. However, the activity of this antimicrobial should continue to be monitored because a slight but continuous increase in the level of non-susceptibility to vancomycin has been found by many studies all around the world [15].

The use of topical agents to treat SSTI is common and has advantages over systemic therapy in terms of side effects and cost effectiveness; however, it promotes the development of resistant strains. Fusidic acid and mupirocin are the most commonly used topical antibiotics worldwide. Many health care practitioners prescribe mupirocin to treat SSTI and to eradicate nasial carriage of *S. aureus* in case of recurrent infections, particularly MRSA. In a nationwide study in the USA, high-level resistance to mupirocin was found in less than 5% of MRSA isolates recovered from the nares and blood [24]. High level mupirocin resistance has been reported to be relatively rare, ranging between 1–5% of MRSA isolates from hospitalized adult populations in North America and Europe [25,26,27,28]. However, higher prevalences (13–45%) have been reported from single center studies [25,29,30]. Lower resistance rates were reported by other USA studies [31,32]. There is a strong association between prior mupirocin exposure and subsequent resistance [29,33,34]. Mupirocin resistance has several implications because it has been associated with resistance to systemic antibiotics, such as clindamycin [31,35]. In the USA, 6.8% of multidrug resistant MRSA isolates demonstrated high-level resistance to mupirocin [31,36], whereas in Europe mupirocin resistance has been reported to be significantly higher in MRSA strains [37,38]. Mupirocin resistance may also aid in the spread of multidrug resistance through coselection of other resistance genes [29]. High-level resistance to mupirocin was found to be low (<1.1%) in a nationwide study in Greece [12]. In our study, we were not able to assess for full susceptibility (<1 mg/L) or high-level resistance (>256 mg/L) to mupirocin because antimicrobial susceptibility testing was performed with the disk diffusion method. Nevertheless, we were able to exclude high-level resistance for 87.3% of our isolates, which suggests that mupirocin remains useful for at least short-term nasal decolonisation of staphylococci in Greece. Resistance to commensal *S. aureus* in a large study from nine European countries revealed low resistance to topical antibiotics, averaging 0.4% for mupirocin and 2.8% for fusidic acid [39]. However, resistance to fusidic acid is high in our study and others in Greece [12], reaching 88% in MRSA [14], possibly due to its extensive use as a topical agent, which makes it totally unreliable in the topical treatment of SSTI.

Existing guidelines do not publish a specific recommendation concerning the application of MRSA screening. Both culture-based methods and polymerase chain reaction (PCR) methods have become widely accepted in applied MRSA diagnostics [40]. The choice of the most appropriate screening method for MRSA is influenced by cost, turn-around time and performance characteristics. One of the major limitations of our study is that the molecular typing that would permit identification of specific clones could not be performed due to the high cost.

Systemic antibiotics in Greece are widely prescribed, guidelines commonly are not followed and patients often acquire topical antibiotics, like mupirocin or fucidic acid without prescription. Therefore, the extensive resistance of *S. aureus* to antibiotics is not surprising and can be easily explained by the aforementioned practices. Given that travelling has become easy and Greece is a popular tourist destination, resistant strains may not be restricted to one area and one single country, and may easily spread to other countries.

## 5. Conclusions

It has been suggested that prescription patterns should be modified if more than 10–15% of MRSA isolates become resistant to a particular antimicrobial agent; however, cycling strategies may not be optimal [23,41]. Special attention is required to prescribing the appropriate antibiotic therapeutic regimens, particularly for MRSA, and the present study providing data on the susceptibility of *S. aureus* may be useful for guiding antibiotic treatment.

## Figures and Tables

**Table 1:** Resistance of *Staphylococcus aureus* isolates to different antibiotics throughout the six-year study period.

Resistance	Total (*n* = 2096)	Period 1 (*n* = 612)	Period 2 (*n* = 477)	Period 3 (*n* = 293)	Period 4 (*n* = 714)
Any	1845 (88.0%)	534 (87.3%)	423 (88.7%)	256 (87.4%)	632 (88.5%)
PEN G	1507 (71.9%)	429 (70.1%)	314 (65.8%)	225 (76.8%)	539 (75.5%)
FOX	440 (21.0%)	125 (20.4%)	138 (28.9%)	4 (1.4%)	173 (24.2%)
CMN	379 (18.1%)	109 (17.8%)	81 (17.0%)	52 (17.7%)	137 (19.2%)
ERY	718 (34.3%)	194 (31.7%)	183 (38.4%)	89 (30.4%)	252 (35.3%)
FUS	480 (22.9%)	126 (20.6%)	132 (27.7%)	72 (24.6%)	150 (21.0%)
GEN	8 (0.4%)	1 (0.2%)	7 (1.5%)	0	0
MNO	13 (0.6%)	2 (0.3%)	6 (1.3%)	3 (1.0%)	2 (0.3%)
MUP	266 (12.7%)	80 (13.1%)	64 (13.4%)	29 (9.9%)	93 (13.0%)
RIF	25 (1.2%)	4 (0.7%)	12 (2.5%)	4 (1.4%)	5 (0.7%)
SXT	96 (4.6%)	32 (5.2%)	24 (5.0%)	4 (1.4%)	36 (5.0%)
TEC	22 (1.0%)	2 (0.3%)	14 (2.9%)	4 (1.4%)	2 (0.3%)
TET	359 (17.1%)	99 (16.2%)	79 (16.6%)	53 (18.1%)	128 (17.9%)

No resistant isolates could be found in vancomycin PEN G: Benzylpenicillin, FOX: Cefoxitin, CMN: Clindamycin, ERY: Erythromycin, FUS: Fusidic acid, GEN: Gentamycin, MNO: Minocycline, MUP: Mupirocin, RIF: Rifampicin, SXT: Trimethoprim-Sulfamethoxazole, TEC: Teicoplanin, TET: Tetracycline.

**Table 2:** Resistance of methicillin-resistant *S. aureus* (MRSA) isolates to different antibiotics.

Resistance	MSSA (*n* = 1629)	MRSA (*n* = 440)
CMN	208 (12.6%)	171 (38.9%)
ERY	453 (27.4%)	265 (60.2%)
FUS	279 (16.8%)	201 (45.7%)
MNO	6 (0.4%)	7 (1.6%)
MUP	188 (11.4%)	78 (17.7%)
RIF	14 (0.8%)	11 (2.5%)
SXT	41 (2.5%)	55 (12.5%)
TEC	11 (0.7%)	11 (2.5%)
TET	203 (12.3%)	156 (35.5%)
GEN	5 (0.3%)	3 (0.7%)

No resistant isolates could be found in vancomycin CMN: Clindamycin, ERY: Erythromycin, FUS: Fusidic acid, MNO: Minocycline, MUP: Mupirocin, RIF: Rifampicin, SXT: Trimethoprim-Sulfamethoxazole, TEC: Teicoplanin, TET: Tetracycline, GEN: Gentamycin.

**Table 3:** Resistance of *S. aureus* isolates to different antibiotics, by location of skin lesion.

Resistance	Location			
	Limbs (*n* = 272)	Trunk (*n* = 150)	Face (*n* = 73)	Unknown (*n* = 1601)
Any	239 (87.9%)	132 (88.0%)	65 (89.0%)	1409 (88.0%)
PEN G	201 (73.9%)	116 (77.3%)	60 (82.2%)	1130 (70.6%)
FOX	71 (26.1%)	40 (26.7%)	13 (17.8%)	316 (19.7%)
CMN	67 (24.6%)	24 (16.0%)	9 (12.3%)	279 (17.4%)
ERY	103 (37.9%)	58 (38.7%)	23 (31.5%)	534 (33.4%)
FUS	52 (19.1%)	30 (20.0%)	14 (19.2%)	384 (24.0%)
GEN	0	0	0	8 (0.5%)
MNO	0	0	0	13 (0.8%)
MUP	28 (10.3%)	29 (19.3%)	19 (26.0%)	190 (11.9%)
RIF	2 (0.7%)	0	1 (1.4%)	22 (1.4%)
SXT	14 (5.1%)	16 (10.7%)	2 (2.7%)	64 (4.0%)
TEC	0	0	0	22 (1.4%)
TET	47 (17.3%)	22 (14.7%)	10 (13.7%)	280 (17.5%)

No resistant isolates could be found in vancomycin PEN G: Benzylpenicillin, FOX: Cefoxitin, CMN: Clindamycin, ERY: Erythromycin, FUS: Fusidic acid, GEN: Gentamycin, MNO: Minocycline, MUP: Mupirocin, RIF: Rifampicin, SXT: Trimethoprim-Sulfamethoxazole, TEC: Teicoplanin, TET:Tetracycline.

**Table 4:** Resistance of *S. aureus* isolates to different antibiotics in outpatients and patients from the Leg Ulcer Unit.

Resistance	Outpatients (*n* = 1766)	Leg Ulcer Unit (*n* = 86)
Any	244 (87.9%)	79 (91.9%)
PEN G	1440 (71.6%)	67 (77.9%)
FOX	429 (21.3%)	11 (12.8%)
CMN	367 (18.3%)	12 (14.0%)
ERY	689 (34.3%)	29 (33.7%)
FUS	467 (23.2%)	13 (15.1%)
GEN	8 (0.4%)	0
MNO	13 (0.6%)	0
MUP	260 (12.9%)	6 (7.0%)
RIF	25 (1.2%)	0
SXT	94 (4.7%)	2 (2.3%)
TEC	22 (1.1%)	0
TET	358 (17.8%)	1 (1.2%)

No resistant isolates could be found in vancomycin PEN G: Benzylpenicillin, FOX: Cefoxitin, CMN: Clindamycin, ERY: Erythromycin, FUS: Fusidic acid, GEN: Gentamycin, MNO: Minocycline, MUP: Mupirocin, RIF: Rifampicin, SXT: Trimethoprim-Sulfamethoxazole, TEC: Teicoplanin, TET:Tetracycline.
